# Analysis of Glucosinolate Content and Metabolism Related Genes in Different Parts of Chinese Flowering Cabbage

**DOI:** 10.3389/fpls.2021.767898

**Published:** 2022-01-17

**Authors:** Xianjun Feng, Jiajun Ma, Zhiqian Liu, Xuan Li, Yinghua Wu, Leiping Hou, Meilan Li

**Affiliations:** ^1^College of Horticulture, Shanxi Agricultural University, Taigu, China; ^2^Collaborative Innovation Center for Improving Quality and Increasing Profits of Protected Vegetables in Shanxi, Taigu, China; ^3^Agriculture Victoria Research, Department of Jobs, Precincts and Regions, AgriBio, Bundoora, VIC, Australia

**Keywords:** Chinese flowering cabbage, organ, glucosinolate, RNA-Seq, gene

## Abstract

Glucosinolates (GSLs) are important secondary metabolites that play important defensive roles in cruciferous plants. Chinese flowering cabbage, one of the most common vegetable crops, is rich in GSLs and thus has the potential to reduce the risk of cancer in humans. Many genes that are involved in GSL biosynthesis and metabolism have been identified in the model plant *Arabidopsis thaliana*; however, few studies investigated the genes related to GSL biosynthesis and metabolism in Chinese flowering cabbage. In the present study, the GSL composition and content in three different organs of Chinese flowering cabbage (leaf, stalk, and flower bud) were determined. Our results showed that the total GSL content in flower buds was significantly higher than in stalks and leaves, and aliphatic GSLs were the most abundant GSL type. To understand the molecular mechanisms underlying the variations of GSL content, we analyzed the expression of genes encoding enzymes involved in GSL biosynthesis and transport in different tissues of Chinese flowering cabbage using RNA sequencing; the expression levels of most genes were found to be consistent with the pattern of total GSL content. Correlation and consistency analysis of differentially expressed genes from different organs with the GSL content revealed that seven genes (*Bra029966*, *Bra012640*, *Bra016787*, *Bra011761*, *Bra006830*, *Bra011759*, and *Bra029248*) were positively correlated with GSL content. These findings provide a molecular basis for further elucidating GSL biosynthesis and transport in Chinese flowering cabbage.

## Introduction

Chinese flowering cabbage (*Brassica campestris* L. ssp. *chinesis* var. *utilis* Tsen et Lee), also known as choy sum, is a variant of a *Brassica rapa* subspecies and belongs to the cruciferous family. It is an annual plant and the tender stalks and flower buds are the main edible organs. Because of the unique flavor and taste of cruciferous plants and its tenderness, Chinese flowering cabbage enjoys the reputation of “the crown of vegetables.” It is rich in nutrients such as proteins and vitamin C, and secondary metabolites such as glucosinolates (GSLs), which are beneficial for human health (Johnson, 2018). Particularly, the flower stalks and buds have the highest nutritional content; therefore, it is popular among consumers (Zou et al., 2021).

GSLs are secondary metabolites rich in nitrogen and sulfur anions. They are divided into three categories based on their side chain structures: aliphatic, indole, and aromatic GSLs (Mithen, 2001; Brader et al., 2006). GSLs play an important defensive role in cruciferous plants against the attacks of fungi and insects. Some GSLs not only possess anti-cancer properties but also determine the flavor, taste, and quality of the plants (Ishida et al., 2014). The GSLs enriched in Chinese flowering cabbage may promote human health and reduce the risk of cancer (Cartea and Velasco, 2008).

The composition and content of GSLs vary not only between different plant species but also between different parts of the same plant. The biosynthesis of GSLs in leaves, inflorescences, seeds, and roots of plants is more active at the young stage than at the mature stage. As the plant grows, the GSL content decreases in the vegetative organs, but increases in the seeds (Nour-Eldin and Halkier, 2009). Analysis of GSLs in different organs of pak choi showed that roots had a significantly higher GSL content than leaves (Zhu et al., 2013).

In *Arabidopsis thaliana*, the expression profiles of GSL biosynthesis genes were analyzed in different tissues and organs and at different developmental stages. It was found that the genes associated with *Arabidopsis* GSL biosynthesis were mainly expressed in vegetative tissues, and their expression levels in storage organs were lower (Du et al., 2016). Also, in *Arabidopsis thaliana*, methylthioalkyl malate synthase genes *MAM1* and *MAM3* displayed the highest expression in the vegetative growth stage, whereas their expression levels in flowers and fruits were relatively low, although the GSL content was higher in flowers and fruits (Redovniković et al., 2012). In Chinese kale, the analysis of genes involved in GSL synthesis in different parts and different developmental stages revealed a higher expression in the cotyledons, leaves, and stalks than in flowers and seeds, and a higher expression in mature leaves compared with young leaves (Yin, 2016). Both CYP83B and SUR1 are involved in the synthesis of the core structure in the second step of GSL biosynthesis. SUR1 can convert S-alkyl-thiohydroxamates into thiohydroximate, and then form the core structure of GSL (Sønderby et al., 2010). CYP83B1 is related to acetaldoxime metabolizing enzyme, which can convert acetaldoxime into GSLs (Naur et al., 2003). In papaya, the relative expression levels of cytochrome P450 *CYP83B* and superroot (*SUR1*) genes were higher in rhizomes than in leaves (Li, 2011).

Until now, there have been few studies on the accumulation and transport of GSLs in Chinese flowering cabbage. In this study, the GSL composition and content in different organs of Chinese flowering cabbage were determined, and the expression patterns of genes involved in GSL metabolism and transport was analyzed using transcriptome sequencing. These findings provide a theoretical basis for using molecular breeding to improve the quality of Chinese flowering cabbage varieties.

## Materials and Methods

### Plant Materials and Cultivation

A late-maturing variety of Chinese flowering cabbage, “Youlv 802,” was used as the research material; the seeds were purchased from the Guangzhou Academy of Agricultural Sciences. Seeds were sown in rows in a plastic greenhouse on Sep. 15th, 2019; the seedlings were thinned to a density of 12 cm × 12 cm between plants and were managed regularly.

### Sample Collection

Chinese flowering cabbage was sown in soil rich in organic matter in the greenhouse. When it reached the harvest standard, ten plants at the same growth stage (the height of the main stalk was equal to the tip of the leaf and the first flower was visible) were randomly selected. Then, approximately 5 g each of leaves, stalks and flower buds were sampled from these plants. A sub-sample of approximately 2 g was rapidly frozen in liquid nitrogen and stored at –80^°^C for transcriptome sequencing; the remaining samples (approximately 3 g) were stored at –30^°^C for determination of GSL content. Both GSL measurement and transcriptome sequencing were performed with three replicates. The leaf samples were labeled as L1, L2, and L3; the stalk samples were labeled as S1, S2, and S3; and the flower bud samples were labeled as B1, B2, and B3.

### Determination of Glucosinolate Content

Fresh leaves, stalks and flower buds were cut into small pieces and then freeze-dried, crushed, sieved, and stored at –30^°^C before GSL extraction. GSL extraction was performed using the method described by Zang et al. (2015).

The GSL content was determined by HPLC as described by Zang et al. (2015). Chromatographic separation was achieved using an Agilent 1200 UPLC system (Agilent Technologies, Inc., Santa Clara, United States) with a Prontosil ODS2 column (250 × 4 mm, 5 μm, Bischoff, Germany). The mobile phase consisted of ultrapure water and acetonitrile (Tedia, United States) and the following gradient elution program was adopted: 0–20% acetonitrile (0–32 min), then 20% acetonitrile (32–38 min), followed by 20–100% acetonitrile (38–43 min). The flow rate was 1.3 mL/min.

Individual glucosinolates were identified by LC-MS analysis. Amounts of glucosinolates were determined with sinigrin as an internal standard and based on the response factors (ISO9167-1) of each compound relative to sinigrin. The glucosinolate content was expressed as μmol⋅100g^–1^dry weight (DW).

### Transcriptome Sequencing

Transcriptome sequencing was commissioned to Wuhan Metroville Biotechnology Co., Ltd. After the extracted RNA passed the quality assessment, approximately 3 μg RNA from each sample was used to construct the cDNA library. Total RNA was extracted by RNeasy Plant Mini Kit (QIAGEN, 74903) according to the manufacturer’s instructions. The mRNA in the total RNA was first enriched using magnetic beads with Oligo (dT). After the mRNA was broken into short fragments by adding fragmentation buffer, the first-strand cDNA was synthesized using six-base random primers, and double-stranded cDNA was synthesized by adding buffer, dNTPs and DNA polymerase I. The double-stranded cDNA was then purified using AMPure XP beads, followed by end repair and sequencing junction ligation. Fragment size selection was performed using AMPure XP beads and cDNA libraries were constructed by PCR amplification. Finally, the sequencing libraries were sequenced using the Illumina MiSeq platform.

Raw reads obtained for each sample after sequencing were subjected to remove primer and reads with adapters. Low-quality reads were filtered out; the clean reads consisted of>80% base pairs with a *Q*-value ≥ 30. The clean reads were aligned with TopHat software using the Brassica Database^1^ as the reference and were assembled into contigs. Using paired-end joining and gap filling, the contigs were assembled and clustered to obtain unique reads. The saturation of reads in each library was evaluated by comparing the number of identified genes vs. total reads.

The transcriptome sequencing data from this study have been deposited in the NCBI SRA database and are accessible through accession number PRJNA717318.^2^ The corresponding sequence data are referenced to BioSample accessions SAMN18497959 (for L1), SAMN18497960 (for L2), SAMN18497961 (for L3), SAMN18497962 (for S1), SAMN18497963 (for S2), SAMN18497964 (for S3), SAMN18497965 (for B1), SAMN18497966 (for B2), and SAMN18497967 (for B3).

### Gene Expression and Functional Annotation Analysis

Gene expression levels were measured as fragments per kilobase of transcript per million mapped reads (FPKM). Differentially expressed genes (DEGs) between different organs were identified using DESeq2 software. A false discovery rate (FDR) < 0.01 and fold change (FC) ≥ 2 was considered as the threshold of differential gene expression. The groups were compared in pairs [leaf (L) vs. stalk (S), L vs. flower bud (B), and S vs. B], and gene ontology (GO) analysis and Kyoto Encyclopedia of Genes and Genomes (KEGG) pathway analysis were performed for all DEGs.

### Quantitative Reverse Transcription PCR Analysis

Eight genes were randomly selected for quantitative reverse transcription PCR (qRT-PCR) analysis in different tissues of Chinese flowering cabbage. The online tool Primer3 was used to design specific primers for the eight genes, and the Chinese flowering cabbage β*-actin* gene was used as the reference gene. The primers are shown in Supplementary Table 1. After RNA extraction, cDNA synthesis by reverse transcription was performed using a PrimeScript RT Reagent Kit (TaKaRa, RR037A) and qRT-PCR was performed using TB Green Premix Ex Taq II (TaKaRa, RR820A). The reaction conditions of the 20 μl reaction system were as follows: 94^°^C for 30 s; 40 cycles of 94^°^C for 30 s, 55^°^C for 30 s, and 72^°^C for 30 s. The PCR reactions were performed on an ABI 7500 real-time PCR system; each reaction was performed with three technical replicates, and the relative expression level was calculated using the 2^–ΔΔCT^ method (Shang et al., 2017). The qRT-PCR verification figure is drawn according to the format of Li et al. (2020).

## Results

### Determination of the Composition and Contents of Glucosinolates in Different Parts of Chinese Flowering Cabbage

We analyzed the GSL composition of leaves, stalks, and flower buds of Chinese flowering cabbage. Seven different GSLs were detected in the three organs, including three aliphatic GSLs (progoitrin, gluconapin, and glucobrassicanapin), three indole GSLs (4-OH-glucobrassicin, glucobrassicin, and 4-methoxy-glucobrassicin), and one aromatic GSL (gluconasturtiin) (Table 1).

**TABLE 1:** Composition and content (μmol/100 g) of glucosinolates in different parts of Chinese flowering cabbage.

Glucosinolate type	Systematic name	Leaf	Stalk	Bud
Total glucosinolates	_	12.73 ± 0.18c	16.04 ± 0.06b	37.92 ± 0.11a
Aliphatic glucosinolate	Progoitrin	3.62 ± 0.24c	4.08 ± 0.04b	7.61 ± 0.08a
	Gluconapin	5.60 ± 0.22b	5.84 ± 0.06b	16.15 ± 0.93a
	Glucobrassicanapin	1.49 ± 0.27c	3.66 ± 0.24b	10.67 ± 0.09a
Indole glucosinolate	4-Hydroxy-Glucobrassicin	0.45 ± 0.01c	0.53 ± 0.03b	0.62 ± 0.01a
	Glucobrassicin	0.37 ± 0.01c	0.42 ± 0.01b	0.95 ± 0.05a
	4-Methoxy-Glucobrassicin	0.33 ± 0.01b	0.28 ± 0.01b	0.51 ± 0.06a
Aromatic glucosinolate	Gluconasturtiin	0.87 ± 0.02b	1.23 ± 0.03a	1.41 ± 0.04a

*Data are mean ± standard deviation. Significant differences were analyzed using Duncan’s multiple range tests by software package SPSS. Different letters indicate a significant difference (P ≤ 0.05).*

The total GSL content of leaves was the lowest, only 12.73 μmol/100 g, and that of stalks was higher (16.04 μmol/100 g). The highest GSL content was found in flower buds (37.92 μmol/100 g).

Overall, the aliphatic GSL content was the highest, whereas indole GSL and aromatic GSLcontents were much lower. A large inter-organ difference was also observed for all three classes of GSLs. Flower buds showed an aliphatic GSL content 3.21 and 2.53 times that in leaves and stalks, respectively. The indole GSL content in flower buds was 1.80 times that in leaves and 1.69 times that in stalks. The aromatic GSL content in flower buds was not significantly different from that in stalks (1.14 times), but was significantly higher than that in leaves (1.62 times).

### Transcriptomic Analysis

#### Evaluation of Sequencing Results

To analyze the gene expression in three different organs of Chinese flowering cabbage, a total of nine libraries, i.e., three replicate libraries for each of leaf (L), stalk (S), and flower bud (B), were constructed. The summary results of high-throughput sequencing data for each library are shown in Supplementary Table 2. The number of reads in the nine libraries was more than 40 million. After contaminated and low-quality sequences were filtered out of the original data, >96% of the reads obtained for subsequent analysis were high-quality reads, implying that the quality of the sequencing data was satisfactory.

The high-quality clean reads obtained from each library were compared with the genome database in Brassica Database (see text footnote 1). The proportion of reads mapped to the reference genome from each library was >88%, and this high match rate indicated that the sequencing results and the selected reference genome were reliable and could be used for subsequent analysis.

#### Sample Correlation Analysis

The repeatability between biological replicates reflects data reliability, and the Pearson correlation coefficient *R* is an evaluation index for the correlation level. Generally, *R* between biological replicates must be at least >0.8 (Supplementary Figure 1). The three biological replicates of the three different organs, leaf (L), stalk (S), and flower bud (B), were compared in pairs. The correlation coefficient of the sample pairs was>0.85, suggesting that the three biological replicates in the experiment had a high degree of similarity, and the transcriptome data obtained was reliable.

#### Verification of RNA-Sequencing Data by Quantitative Reverse Transcription PCR

**FIGURE 1:**
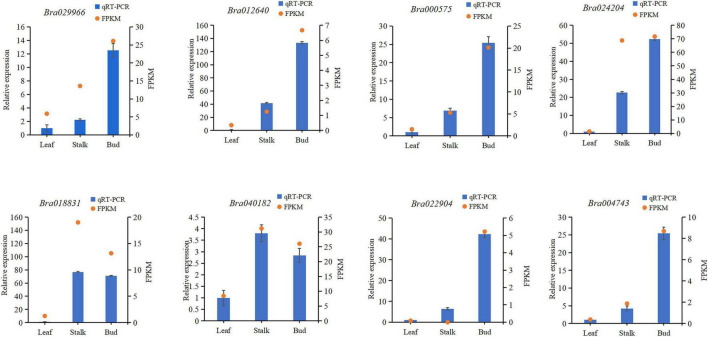
Comparison of quantitative reverse transcription PCR results with gene expression levels determined by RNA sequencing. FPKM, fragments per kilobase of transcript per million mapped reads.

To verify the reliability of the RNA sequencing (RNA-Seq) results, eight DEGs from three different organs of Chinese cabbage were randomly selected (*Bra029966, Bra012640, Bra040182, Bra000575, Bra022904, Bra024204, Bra004743*, and *Bra018831*) for qRT-PCR verification. The total RNA from the three organs was extracted for qRT-PCR experiments, and the qRT-PCR results were compared with the transcriptome sequencing data. It was found that the FPKM values of all genes were consistent with the relative expression levels (Figure 1), indicating that the transcriptome sequencing results were reliable and could be used to assess the magnitude of gene expression changes.

#### Expression Analysis of Genes Encoding Enzymes Related to Glucosinolate Metabolism

**FIGURE 2:**
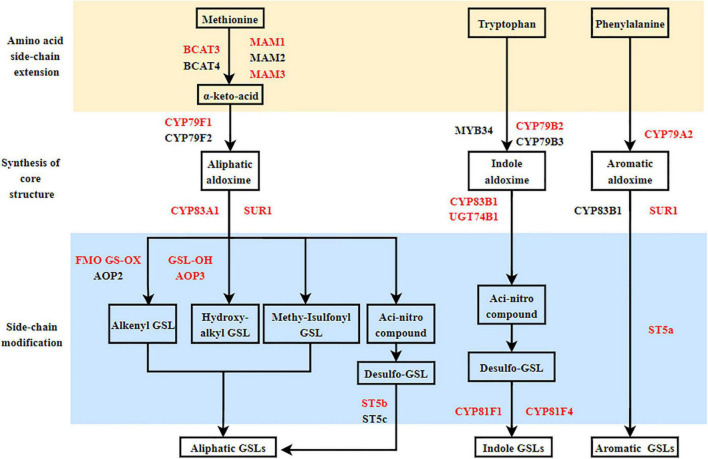
Proposed glucosinolate (GSL) biosynthesis pathways in Chinese flowering cabbage based on the transcriptome and GSL quantification data. Red letter: Key enzymes.

The GSL biosynthetic pathway is divided into three parts: (1) amino acid side-chain extension (2) core structure formation, and (3) side-chain modification (Figure 2). There are nine genes encoding the enzymes that regulate aliphatic GSL biosynthesis, including the genes involved in amino acid side-chain extension (*BCAT3*, *MAM1*, and *MAM3*), core structure formation (*CYP79F1*, *CYP83A1*, and *ST5b*) and side-chain modification (*FMO FS-OX*, *GSL-OH*, and *AOP3*); five genes involved in indole GSL synthesis, including *CYP79B2*, *CYP81F1/F4*, *CYP83B1*, and *UGT74B1*; three genes involved in aromatic GSL synthesis, including *CYP79A2*, *SUR1*, and *ST5a*. In addition, two genes, *GTR1* and *GTR2* are known to be involved in GSL transport.

On the basis of *Arabidopsis* genes related to GSL metabolism, we identified 59 and 7 homologous genes associated with GSL synthesis and transport, respectively, on the Chinese cabbage website (Table 2). Expression analysis of these genes in three different organs of Chinese flowering cabbage showed that most of the GSL synthesis genes were upregulated in “S vs. B” comparison, consistent with the pattern observed in GSL content (Table 1). As GSLs are mainly synthesized in leaves, the highest expression of GSL biosynthesis genes was found in leaves, followed by flower buds and stalks (Table 2). The highest expression of the transporter genes *Bra010111* and *Bra029248* was also detected in the leaves, presumably allowing efficient transport of the synthesized GSLs to other organs.

**TABLE 2:** Expression of genes encoding enzymes involved in glucosinolate metabolism.

Glucosinolate type	Enzyme	Coding gene	FPKM			Expression pattern		
			Leaf	Stalk	Bud	L vs. S	L vs. B	S vs. B
Aliphatic glucosinolate biosynthesis	BCAT3	*Bra029966*	5.91 ± 0.83	13.62 ± 4.91	26.10 ± 3.43	↑	↑	↑
		*Bra017964*	33.23 ± 2.33	30.18 ± 4.10	31.18 ± 2.00	↓	↓	↑
		*Bra018831*	1.26 ± 0.48	19.01 ± 0.61	13.15 ± 0.81	↑	↑	↓
	BCAT4	*Bra022448*	36.95 ± 0.79	12.87 ± 0.28	25.62 ± 0.76	↓	↓	↑
		*Bra001761*	19.59 ± 3.01	5.26 ± 0.86	8.27 ± 0.84	↓	↓	↑
	MAM1	*Bra029355*	34.05 ± 3.07	8.42 ± 0.81	17.64 ± 4.27	↓	↓	↑
		*Bra013011*	0.00	0.06 ± 0.01	0.00	↑	-	↓
		*Bra013009*	21.13 ± 0.04	3.64 ± 0.02	19.77 ± 1.03	↓	↓	↑
		*Bra021947*	0.00	0.00	0.37 ± 0.03	-	↑	↑
	MAM3	*Bra029356*	0.00	0.04 ± 0.04	0.13 ± 0.04	↑	↑	↑
		*Bra012640*	0.35 ± 0.05	1.25 ± 0.57	6.67 ± 0.24	↑	↑	↑
		*Bra040182*	8.44 ± 0.64	31.18 ± 3.76	26.04 ± 2.22	↑	↑	↓
		*Bra013007*	0.02 ± 0.01	0.03 ± 0.01	0.02 ± 0.01	↑	-	↓
	CYP79F1	*Bra026058*	48.90 ± 2.61	14.25 ± 1.18	11.35 ± 1.70	↓	↓	↓
	CYP83A1	*Bra032734*	275.31 ± 7.12	51.49 ± 2.79	96.59 ± 7.62	↓	↓	↑
		*Bra016908*	17.64 ± 2.24	14.60 ± 1.71	15.76 ± 0.51	↓	↓	↑
	ST5b	*Bra022904*	0.09 ± 0.02	0.00 ± 0.00	5.22 ± 0.56	↓	↑	↑
		*Bra015938*	33.92 ± 4.23	14.33 ± 2.61	12.87 ± 2.71	↓	↓	↓
		*Bra015936*	0.10 ± 0.02	0.07 ± 0.01	0.19 ± 0.03	↓	↑	↑
		*Bra003818*	0.02 ± 0.01	0.01 ± 0.00	0.04 ± 0.01	↓	↑	↑
		*Bra003817*	0.00	0.00	0.10 ± 0.02	-	↑	↑
		*Bra003726*	0.00	3.69 ± 0.56	0.08 ± 0.01	↑	↑	↓
	ST5c	*Bra025668*	26.56 ± 1.72	2.76 ± 0.24	8.05 ± 0.52	↓	↓	↑
	FMO GS-OX1	*Bra027036*	64.09 ± 6.37	27.38 ± 1.97	59.65 ± 3.93	↓	↓	↑
		*Bra027035*	59.63 ± 5.32	21.67 ± 1.86	52.72 ± 4.06	↓	↓	↑
	FMO GS-OX5	*Bra026988*	28.27 ± 2.66	30.00 ± 3.85	10.52 ± 0.93	↑	↓	↓
		*Bra016787*	0.22 ± 0.04	1.28 ± 0.23	3.46 ± 0.56	↑	↑	↑
	AOP2	*Bra018521*	7.18 ± 0.16	1.25 ± 0.22	6.67 ± 0.20	↓	↓	↑
		*Bra034180*	7.21 ± 0.18	1.52 ± 0.74	4.70 ± 0.21	↓	↓	↑
	GSL-OH	*Bra021670*	0.00	0.02 ± 0.01	0.00	↑	-	↓
		*Bra021671*	0.00	0.01 ± 0.00	0.05 ± 0.01	↑	↑	↑
Indole glucosinolate biosynthesis	CYP79B2	*Bra017871*	7.07 ± 0.88	0.07 ± 0.02	0.83 ± 0.24	↓	↓	↑
		*Bra011821*	27.55 ± 3.95	0.39 ± 0.03	6.40 ± 0.27	↓	↓	↑
		*Bra010644*	0.91 ± 0.09	0.05 ± 0.02	0.41 ± 0.14	↓	↓	↑
	CYP79B3	*Bra030246*	6.43 ± 0.58	0.19 ± 0.07	2.95 ± 0.72	↓	↓	↑
	CYP83B1	*Bra034941*	128.34 ± 17.53	6.47 ± 0.48	37.88 ± 4.43	↓	↓	↑
	CYP81F1	*Bra011762*	10.27 ± 0.39	5.14 ± 0.30	22.54 ± 0.59	↓	↑	↑
		*Bra011761*	0.00	0.00	13.93 ± 3.63	-	↑	↑
	CYP81F2	*Bra006830*		0.00	7.51 ± 0.36	-	↑	↑
		*Bra020459*	0.21 ± 0.06	0.02 ± 0.01	0.05 ± 0.01	↓	↓	↑
		*Bra002747*	0.39 ± 0.18	0.00 ± 0.00	0.18 ± 0.02	↓	↓	↑
	CYP81F4	*Bra011759*	0.00	0.14 ± 0.04	5.53 ± 0.24	↑	↑	↑
		*Bra010598*	1.49 ± 0.55	0.00	0.75 ± 0.47	↓	↓	↑
	UGT74B1	*Bra024634*	38.60 ± 2.93	10.42 ± 0.53	17.03 ± 0.72	↓	↓	↑
Aromatic glucosinolate	CYP79A2	*Bra009100*	0.08 ± 0.01	0.00	0.53 ± 0.19	↓	↑	↑
		*Bra028764*	0.00	0.00	0.14 ± 0.08	-	↑	↑
		*Bra005870*	0.00	0.00	0.12 ± 0.03	-	↑	↑
	SUR1	*Bra036703*	53.93 ± 7.12	27.02 ± 1.36	9.35 ± 0.49	↓	↓	↓
		*Bra036490*	103.07 ± 16.31	20.08 ± 1.57	38.48 ± 3.53	↓	↓	↑
		*Bra024204*	1.42 ± 0.96	68.8 ± 5.58	71.74 ± 8.23	↑	↑	↑
	ST5a	*Bra015935*	12.03 ± 0.79	1.06 ± 0.65	3.35 ± 0.23	↓	↓	↑
		*Bra008132*	127.85 ± 17.66	21.28 ± 1.98	36.96 ± 2.92	↓	↓	↑
Transport	GTR2	*Bra010111*	17.10 ± 4.20	17.03 ± 4.59	6.52 ± 0.96	↓	↓	↓
		*Bra029248*	15.38 ± 4.29	13.28 ± 1.06	4.04 ± 0.77	↓	↓	↓
		*Bra035885*	0.39 ± 0.15	2.49 ± 0.81	0.29 ± 0.04	↑	↓	↓
		*Bra035886*	0.01 ± 0.01	0.19 ± 0.11	0.01 ± 0.01	↑	-	↓
	GTR1	*Bra018096*	45.72 ± 4.40	32.90 ± 1.29	20.52 ± 3.87	↓	↓	↓
		*Bra033782*	0.22 ± 0.10	0.35 ± 0.18	1.54 ± 0.31	↑	↑	↑
		*Bra019521*	2.61 ± 0.45	4.25 ± 0.35	5.27 ± 0.15	↑	↑	↑

*—, uniformity; FPKM, fragments per kilobase of transcript per million mapped reads; S, Stalk; L, Leaf; B, Bud.*

Branched-chain amino acid transaminase (BCAT) and methylthioalkyl malate synthase (MAM) play a key role in catalyzing the synthesis of aliphatic GSLs. Transcriptome analysis showed that *BCAT3* (*Bra029966*) and *MAM3* (*Bra012640* and *Bra029356*) had the highest expression in flower buds and the lowest expression in leaves (Table 2). *Bra016787* and *Bra021671*, encoding flavin monooxygenase (FMO GS-OX5) and glucosinolate hydroxylase (GSL-OH), respectively, were all up-regulated in “L vs. S,” “L vs. B,” and “S vs. B” comparisons. However, *Bra021947* and *Bra003817*, encoding MAM1 and sulfotransferase 5b (ST5b), respectively, were not expressed in leaves and stalks, but their expression was also upregulated in flower buds. The expression of *Bra022904*, *Bra015936*, and *Bra003818*, encoding ST5b, was downregulated in “L vs. S” comparison, but was significantly upregulated in flower buds as compared to stalks and leaves. These results are consistent with the GSL content pattern (Table 1).

Among the genes involved in the regulation of indole GSL synthesis, *Bra011759*, encoding a cytochrome P450 homolog CYP81F4, was significantly upregulated in “L vs. S,” “L vs. B,” and “S vs. B” comparisons, whereas *Bra011761* and *Bra006830*, encoding CYP81F1 and CYP81F2, respectively, were not expressed in leaves and stalks but were significantly expressed in flower buds (Table 2).

Among the genes involved in the regulation of aromatic GSL synthesis, *Bra024204*, encoding carbon-sulfur lyase 1 (SUR1), was upregulated in “L vs. S,” “L vs. B,” and “S vs. B” comparisons. *Bra028764* and *Bra005870*, encoding the cytochrome P450 homolog CYP79A2, were not expressed in leaves and stalks, and their expression was upregulated in flower buds. The expression differences of these genes across different organs were consistent with the significantly higher GSL content in flower buds than in leaves and stalks (Table 1), suggesting that these genes play an important role in the regulation of GSL synthesis and are responsible for the significantly higher GSL content in flower buds as compared to leaves and stalks.

The expression of some genes involved in GSL synthesis changed irregularly (Table 2). Several genes involved in aliphatic GSL synthesis, *Bra017964*, *Bra022448*, and *Bra001761* encoding branched-chain amino acid transaminase (BCAT), *Bra029355* and *Bra013009* encoding MAM1, *Bra032734* and *Bra016908* encoding the cytochrome P450 homolog CYP83A1, *Bra027035* and *Bra027036* encoding FMO GS-OX1, *Bra018521* and *Bra034180* encoding 2-oxoglutarate oxygenase (AOP), showed the highest expression in leaves, lowest expression in stalks, and intermediate expression in flower buds. Four genes that regulate indole GSL synthesis—*Bra017871*, *Bra011821*, and *Bra010644*, which encode CYP79B2, and *Bra034941*, which encodes CYP83B1—were downregulated in “L vs. S” and “L vs. B” comparisons but upregulated in “S vs. B” comparison. Two genes involved in the regulation of aromatic GSL synthesis—*Bra015935* and *Bra008132*, which encode ST5a, showed the highest expression in the leaves.

GSL transporter proteins (GTRs) are involved in GSL transport. Transcriptome analysis (Table 2) showed that the expression of most of the transporter genes was downregulated in “L vs. S” and “S vs. B” comparisons, consistent with the trend in GSL content (Table 1). *Bra010111*, *Bra018096*, and *Bra029248* were all downregulated in “L vs. S,” “L vs. B,” and “S vs. B” comparisons. *Bra035885* and *Bra035886* were downregulated in “S vs. B” comparison. Leaves are the main location of GSL biosynthesis; thus, the downregulation of these genes in the flower buds indicated that the expression level of these transporter genes is the highest in the leaves, which may facilitate the long-distance transport of GSLs out of the leaves, leading to their high accumulation in flower buds.

Furthermore, we also found that the expression of 14 genes encoding GSL synthesis and transporters (including *Bra029966*, *Bra029356*, *Bra012640*, *Bra016787*, *Bra021671*, *Bra011761*, *Bra006830*, *Bra011759*, *Bra028764*, *Bra005870*, *Bra024204*, *Bra010111*, *Bra029248*, and *Bra018096*) was consistent with the levels of GSL contents (Tables 1, 2).

### Identification of Key Genes Related to Glucosinolates Synthesis and Transport

By analyzing the transcriptome data of three organs, 69 genes involved in GSL synthesis and transport with FDR < 0.01 and log_2_ (FC) ≥ 1 were identified as DEGs. In order to screen the key genes involved in GSL synthesis and transport, a correlation analysis between the expression level of DEGs and GSL content was conducted and P ≤ 0.001 was used as the threshold. In total, 16 genes were found to be closely associated with various GSLs (Supplementary Figure 2 and Table 3). There were 5, 14, and 15 DEGs identified in the comparisons “L vs. S,” “L vs. B,” and “S vs. B” comparisons, respectively, with four genes common to all three comparisons. Two genes (*Bra029248* and *Bra033782*) were associated with GSL transport, one gene associated with synthesis of aromatic GSL, and the rest associated with the synthesis of aliphatic and indole GSLs.

**TABLE 3:** Analysis of key differentially expressed genes closely associated with glucosinolate metabolism.

Gene name	Coding enzyme	GO function	FPKM			Log_2_FC		
			Leaf	Stalk	Bud	L vs. S	L vs. B	S vs. B
*Bra029966*	BCAT3	Leucine biosynthetic process	5.91 ± 0.83	13.62 ± 4.91	26.10 ± 3.43	–	1.119	–
*Bra012640*	MAM3	Glucosinolate biosynthetic process	0.35 ± 0.05	1.25 ± 0.57	6.67 ± 0.24	–	3.196	2.114
*Bra004743*	IPMI SSU1	Same as above	0.37 ± 0.21	1.87 ± 0.79	8.69 ± 0.55	1.615	3.514	1.914
*Bra008466*	IMD2	Leucine biosynthetic process	0.03 ± 0.01	0.01 ± 0.01	1.51 ± 0.58	–	4.737	6.638
*Bra022904*	ST5b	Glucosinolate biosynthetic process	0.09 ± 0.02	0.00	5.22 ± 0.56	–	4.771	9.082
*Bra016787*	FMO GS-OX5	Same as above	0.22 ± 0.04	1.28 ± 0.23	3.46 ± 0.56	1.858	2.967	1.119
*Bra011761*	CYP81F1	Indole glucosinolate metabolic process	0.00	0.00	13.93 ± 3.63	–	11.091	11.974
*Bra011762*	CYP81F1	Same as above	10.27 ± 0.39	5.14 ± 0.30	22.54 ± 0.59	–1.702	–	1.818
*Bra006830*	CYP81F2	Same as above	0.00	0.00	7.51 ± 0.36	–	10.179	11.059
*Bra011759*	CYP81F4	Same as above	0.00	0.14 ± 0.04	5.53 ± 0.24	–	9.763	5.079
*Bra009100*	CYP79A2	Glucosinolate biosynthetic process	0.08 ± 0.01	0.00	0.53 ± 0.19	–	–	7.325
*Bra000575*	GRF1	Glucosinolate metabolic process	1.51 ± 0.38	5.28 ± 0.89	20.13 ± 4.37	1.100	2.704	1.620
*Bra035954*	MYB34	Indole glucosinolate biosynthetic process	0.00	0.00	0.56 ± 0.25		5.714	6.591
*Bra033782*	GTR1	Glucosinolate transport	0.22 ± 0.10	0.35 ± 0.18	1.54 ± 0.31		1.817	1.857
*Bra029248*	GTR2	Same as above	15.38 ± 4.29	13.28 ± 1.06	4.04 ± 0.77		–2.927	–2.025
*Bra004836*	BGLU15	Glucosinolate metabolic process	0.02 ± 0.01	1.96 ± 0.64	54.73 ± 4.49	5.732	10.227	4.518

*—, uniformity.*

**FIGURE 3:**
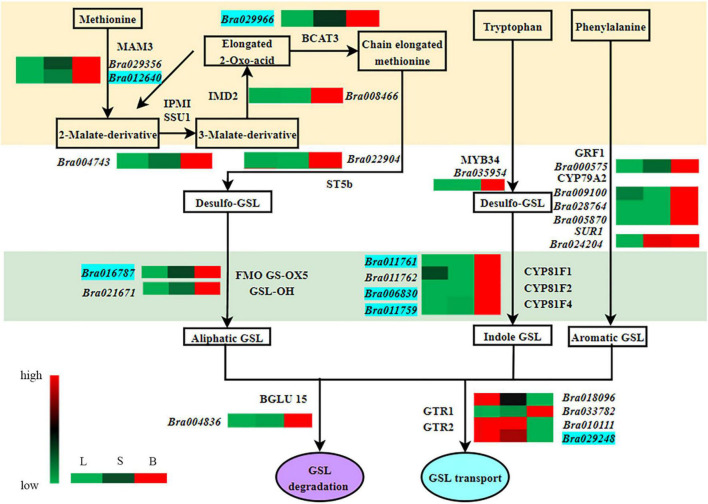
Analysis of differentially expressed genes closely related to glucosinolate metabolism in different organs of Chinese flowering cabbage. L, leave; S, stalk; B, flower buds; Green, black, and red indicate gene expression from low to high. The genes in the blue background are strongly correlated with glucosinolate content.

*Bra029966, Bra012640, Bra004743*, and *Bra008466* encode BCAT3, MAM3, isopropyl malate isomerase (IPMI SSU1) and isopropyl malate dehydrogenase 2 (IMD2), respectively, all of the four enzymes are involved in the first step of GSL biosynthesis. *Bra016787* and *Bra022904* encode FMO GS-OX5 and ST5b, respectively, two enzymes involved in the third step of GSL biosynthesis and functioning as side-chain-modifying enzymes. The expression of these genes was significantly higher in both flower buds and stalks than in leaves (*Bra022904* was not expressed in stalks), which may be the driving force for the enhanced accumulation of GSLs in the bud (Figure 3 and Table 3).

*Bra011761* and *Bra011762*, *Bra006830*, and *Bra011759* encode cytochrome P450 CYP81F1, CYP81F2, and CYP81F4, respectively, which are the key enzymes in the biosynthesis of the core structure of indole GSLs and belong to the CYP81 enzyme family. None of their coding genes, except *Bra011762*, were expressed in leaves. *Bra011761* and *Bra006830* were not expressed in stalks, but both were upregulated in flower buds (Figure 3 and Table 3), which may be responsible for the accumulation of indole GSLs in flower buds.

*Bra000575* and *Bra035954* encode the transcription factors GRF1 and MYB34. GRF1 is involved in many plant processes, including root and leaf growth, flower organ development and resistance to stress (Kim et al., 2003). In addition, GRF1 is associated with the biosynthesis of aromatic GSLs. Its expression was the highest in flower buds and the lowest in leaves (Figure 3 and Table 3). The transcription factor MYB34 regulates the expression of the gene encoding CYP79B2/B3, which catalyzes the conversion of tryptophan to aldoxime and formation of indole GSLs (Celenza et al., 2005). *Bra035954* was not expressed in the leaves and stalks, but was expressed in the flower buds (Figure 3 and Table 3), which may explain the strong accumulation of aromatic and indole GSLs in flower buds.

*Bra009100* encodes CYP79A2, which catalyzes the formation of phenylethylaldoxime from phenylalanine, and finally synthesis of aromatic GSLs. This gene was upregulated in “L vs. B” and “S vs. B” comparisons (Figure 3 and Table 3), consistent with the accumulation of aromatic GSLs in flower buds (Table 1).

*Bra029248* and *Bra033782* encode GTR2 and GTR1, respectively, which belong to the NRT1/PTR protein family. *Bra029248* was downregulated in “L vs. S” and “S vs. B” comparisons (Figure 3 and Table 3) and showed a highly significant negative correlation with aliphatic GSL content. This indicated that the rate of GSL transport was higher in the leaves than in the shoots and buds, leading to the higher level of GSLs in flower buds. *Bra033782* was upregulated in “L vs. S” and “S vs. B” comparisons, in contrast to the *Bra029248* expression trend (Figure 3 and Table 3); however, the difference among organs was small, the underlying reason remains to be investigated.

*Bra004836* encodes β-glucosidase (BGLU15), an enzyme that degrades GSLs. The gene was highly expressed in flower buds (Figure 3 and Table 3). Given that the GSL content was the highest in buds, this gene is likely to be associated with bud development rather than GSL degradation.

In summary, our study found a total of 14 key genes involved in GSL biosynthesis and transport and their expression was consistent with GSL content. We also identified 16 DEGs that were closely related to GSL composition and content. Among them, the expression of seven genes—*Bra029966* (BCAT3), *Bra012640* (MAM3), *Bra016787* (FMO GS-OX5), *Bra011761* (CYP81F), *Bra006830* (CYP81F), *Bra011759* (CYP81F) and *Bra029248* (GTR)—was consistent with the content of GSL, implying that these genes may be directly associated with GSL synthesis and transport (Figure 3). Their functions in the biosynthesis and transport of GSLs need to be further confirmed in Chinese flowering cabbage.

## Discussion

The GSL composition and content in plants vary not only between species and growing environments but also between different organs of the same plant (Li et al., 2021). GSLs are first synthesized and accumulated in young leaves. As the plants grow and bloom, the GSL content in leaves decreases as the GSLs are transported to and accumulated in flower buds and seeds (Paul et al., 2003). A previous study showed that the GSL content in *Arabidopsis* was the highest in dormant seeds and germinated seeds, followed by inflorescence, siliques, leaves, roots, and stems (Paul et al., 2003). GSL content in fleshy roots and leaves of different varieties of radishes was relatively high but significantly different. GSL content in roots is much higher than that in leaves (Yang et al., 2010). In different varieties of rape, GSL content in the buds was higher than that in the stalk; however, the components of GSLs in both organs were similar, i.e., glucobrassicanapin and progoitrin (Peng et al., 2019). Cruciferous vegetables have high GSL content and similar components in flower buds and stalks. In the present study, the GSL content in three different organs of Chinese flowering cabbage was determined, and seven types of GSLs were detected in all organs; the content in buds was the highest, followed by that in the stalk and leaves, respectively, which is consistent with other research’s findings (Li et al., 2021).

RNA sequencing results suggested that the expression patterns of 14 genes encoding enzymes involved in GSL synthesis and transport were positively correlated with the variation in GSL content, including *Bra029966*, *Bra029356*, *Bra012640*, *Bra016787*, *Bra021671*, *Bra011761*, *Bra006830*, *Bra011759*, *Bra028764*, *Bra005870*, *Bra024204*, *Bra010111*, *Bra029248*, and *Bra018096*; and their homologous genes in *Arabidopsis* encode BCAT3, MAM3, FMO GS-OX5, GSL-OH, CYP81F1, CYP81F2, CYP81F4, CYP79A2, SUR1, and GTR2/1, respectively.

The first three genes are involved in the side-chain extension in the first step of GSL biosynthesis. BCAT3, together with BCAT4 enzyme, catalyzes the deamination and transamination in side-chain extension (Schuster et al., 2006; Knill et al., 2008). The MAM3 enzyme catalyzes all the different cycles of methionine chain extension (Textor et al., 2007). Overexpression of the *Arabidopsis* gene MAM in Chinese cabbage increases the accumulation of gluconapin and glucobrassicanapin (Zang et al., 2008). *Arabidopsis* MAM3 deletion mutants show significant reduction or complete absence of long-chain GSLs (Textor et al., 2007). Therefore, *Bra029966* and *Bra012640* encoding BCAT and MAM enzymes are required for GSL accumulation.

FMO GS-OX5 participates in the last step of GSL synthesis, which is the oxidation process of sulfur on the side chain of aliphatic R. A study on the GSL levels of five loss-of-function mutants revealed that the GSL content of four mutants was significantly reduced after oxidation (Hansen et al., 2007; Li et al., 2008). In another study, two genes homologous to AtFMO GS-OX5 were identified in Chinese cabbage, and such side-chain modification genes were found to be highly expressed in reproductive organs, flower buds, and pods (Nugroho et al., 2020); our results are similar to these findings. Thus, a high expression of genes encoding GS-OX5 promotes the synthesis and accumulation of GSLs in flower buds.

CYP81F1/2/4 is involved in the synthesis of indole GSLs. The upregulation of three genes encoding CYP81F1/2/4 in stalk and flower buds is in agreement with the higher indole GSL content in stalk and buds as compared to leaves. CYP79A2 participates in the synthesis of the core structure in the second step of GSL biosynthesis. The precursor amino acids are catalyzed by the cytochrome P450 CYP79 family of proteins to generate acetaldoxime. Alanine-derived acetaldoxime is catalyzed by CYP79A2 (Wittstock and Halkier, 2000). In our experiment, *Bra028764* and *Bra005870* encoding CYP79A2 were expressed only in the buds, matching the increased aromatic GSL levels in the buds. The C-S lyase SUR1 is involved in the biosynthesis of aliphatic and aromatic GSL; therefore, upregulation of the SUR1-encoding gene increases the GSL content (Mikkelsen et al., 2004).

By analyzing the correlation between the DEGs and GSL type and content in different organs, 16 genes closely related to the synthesis and transport of three GSL classes were identified. Some of the genes have been previously described. *Bra035954*, encoding MYB34, acts as a transcription factor regulating the biosynthesis of indole GSLs (Henning and Tamara, 2014). Through subcellular localization, MYB34 protein was found to be expressed in both the nucleus and cytoplasm. In chinese cabbage, the expression of different genes encoding MYB34 isoforms was different, but all of them showed a higher expression in the seeds (Kim et al., 2013). In the present study, *Bra035954* was expressed only in the flower buds of Chinese flowering cabbage, but not in leaves and stalks, which may explain the higher indole GSL content in flower buds.

*Bra000575* encodes the transcriptional activator GRF1. The homologous gene encoding GRF has been studied in *Arabidopsis*, corn, and rice. GRF takes part in many processes including: cell proliferation, leaves expansion, flower development and so on (Kim et al., 2003). In rice, the expression of OsGIF1 occurred in all tissues at a high level while that of OsGRF1 appeared preferentially only in the stem tips containing shoot apical meristem and younger leaves containing leaf primordium (Lu et al., 2020). Our results showed that the expression of *Bra000575* was the lowest in leaves, followed by stalks and flowers, which was consistent with previous findings and the pattern of GSL content in these organs.

*Bra011759* encodes cytochrome P450 CYP81F4. Analysis of the metabolic profile of *Arabidopsis* CYP81F4 deletion mutant strains found glucobrassicin accumulation (Kosuke et al., 2011). In chinese flowering cabbage, the expression of this gene was the highest in flower buds, but not in the leaves, which may be one of the reasons for the high GSL content in the flower buds.

Expression of *Bra029248*, which encodes GTR2, was the highest in leaves. Research in *Arabidopsis* has found that GSLs are specifically transported from rosette leaves and roots to inflorescences in a GTR1- and GTR2-dependent manner, indicating that GSLs are transported to inflorescences during bolting (Andersen and Halkier, 2014). Our results are consistent with these findings. Another study reported that GSLs synthesized by leaves are transported to flower buds and fruit pods, and then the transported and self-synthesized GSLs are transported to seed embryos (Du and Halkier, 1998; Jiang et al., 2019). This also explains the high levels of GSLs in flower buds and stalks. Clearly, further work is needed to understand the mechanisms of the gene function.

## Conclusion

In this study, the GSL composition and content of three different organs of Chinese flowering cabbage were analyzed. Overall, the content of aliphatic GSLs was the highest, followed by aromatic GSLs and indole GSLs. Across the three organs, the total GSL content in flower buds was significantly higher than that in stalks and leaves, and the total GSL content in leaves was relatively low. Expression analysis of the genes encoding enzymes involved in GSL biosynthesis and transport revealed that the expression levels of most genes followed the same pattern as the total GSL content. This may explain the higher GSL content in flowers buds as compared to leaves. In addition, by analyzing the correlation and consistency between expression of genes associated with GSL metabolism and transport and GSL composition and contents, seven genes (including *Bra029966*, *Bra012640*, *Bra016787*, *Bra011761*, *Bra006830*, *Bra011759*, and *Bra029248*) were found to be closely associated with GSL profile and content. It is suggested that these genes play an important role in GSL synthesis and transport. These findings are valuable for further elucidating the molecular mechanism of GSL synthesis and transport in different organs of Chinese flowering cabbage.

## Data Availability Statement

The datasets presented in this study can be found in online repositories. The names of the repository/repositories and accession number(s) can be found below: https://www.ncbi.nlm.nih.gov/sra/, PRJNA717318.

## Author Contributions

XF and JM performed the experiment, data analysis and prepared the manuscript. ZL, XL, and YW performed the experiments. LH and ML participated in the experimental design. ML conceived the idea and participated in the interpretation of results and preparation of manuscript. All authors read and approved the final manuscript.

## Conflict of Interest

The authors declare that the research was conducted in the absence of any commercial or financial relationships that could be construed as a potential conflict of interest.

## Publisher’s Note

All claims expressed in this article are solely those of the authors and do not necessarily represent those of their affiliated organizations, or those of the publisher, the editors and the reviewers. Any product that may be evaluated in this article, or claim that may be made by its manufacturer, is not guaranteed or endorsed by the publisher.

## Footnotes

1
http://brassicadb.org/brad/

2
https://www.ncbi.nlm.nih.gov/sra/PRJNA717318

## Supplementary Material

Supplementary Figure 1 Correlation heat map of RNA sequencing data across the 3 biological replicates. L, Leaf; S, Stem; B, Bud. Yellow to red in the heat map represents the correlation coefficient from low to high.

Supplementary Figure 2 Heat map of correlation between DEG’s expression levels and glucosinolate content. Blue, white, and red in the heat map represent the correlation index r from low to high.
