# Supplementary figures and images for: Analysis of Glucosinolate Content and Metabolism Related Genes in Different Parts of Chinese Flowering Cabbage
Source: Front Plant Sci. 2022 Jan 17;12:767898. doi: 10.3389/fpls.2021.767898 (PMC8801782; doi:10.3389/fpls.2021.767898)

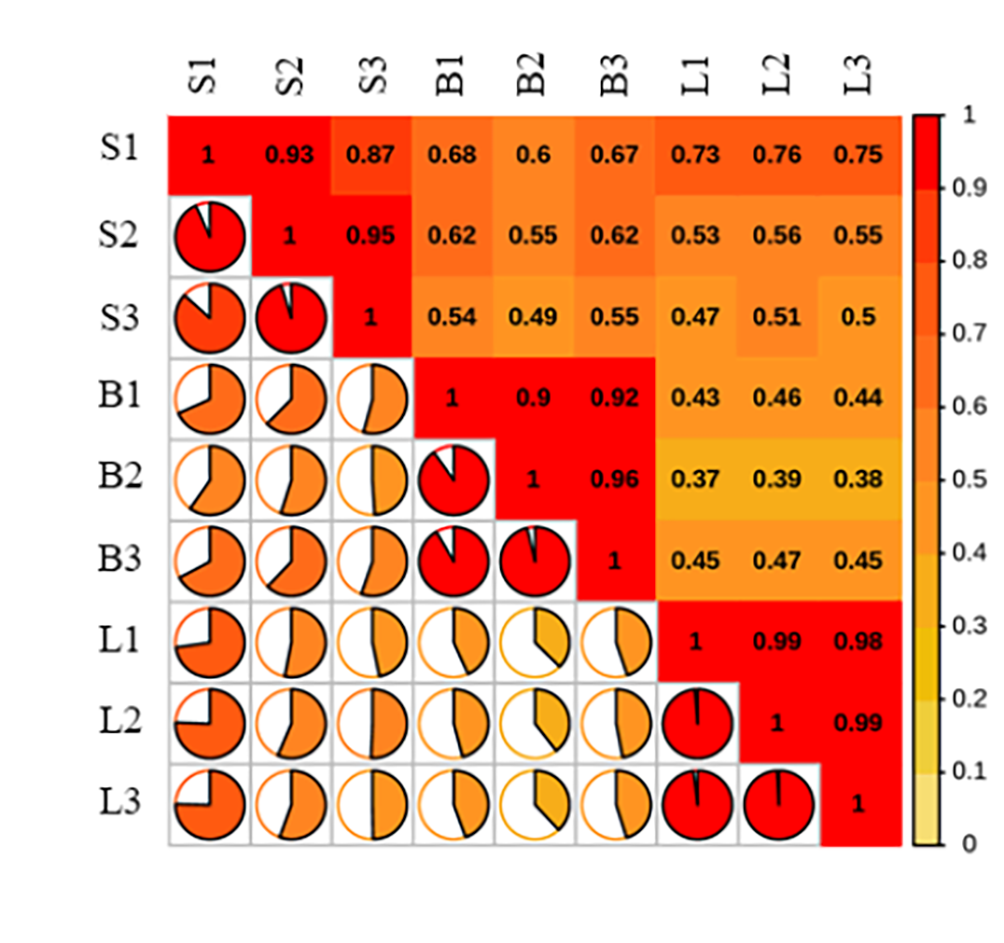

Supplement: Supplementary Figure 1 — Correlation heat map of RNA sequencing data across the 3 biological replicates. L, Leaf; S, Stem; B, Bud. Yellow to red in the heat map represents the correlation coefficient from low to high. [file Image_1.TIF]

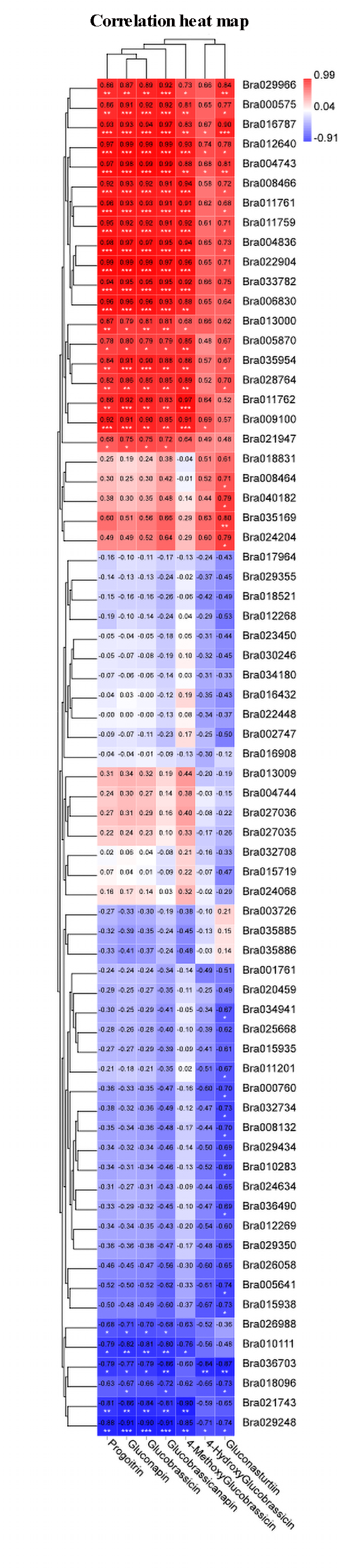

Supplement: Supplementary Figure 2 — Heat map of correlation between DEG’s expression levels and glucosinolate content. Blue, white, and red in the heat map represent the correlation index r from low to high. [file Image_2.TIF]
