# Supplementary material for: Analysis of Glucosinolate Content and Metabolism Related Genes in Different Parts of Chinese Flowering Cabbage
Source: Front Plant Sci. 2022 Jan 17;12:767898. doi: 10.3389/fpls.2021.767898 (PMC8801782; doi:10.3389/fpls.2021.767898)
Supplement: Supplementary file 3 [file Table_1.DOCX]

**Supplementary Table S1** qRT-PCR primer sequences

| gene name | F | R |
| --- | --- | --- |
| *ACTIN* | GTTGCTATCCAGGCTGTTCT | AGCGTGAGGAAGAGCATAAC |
| *Bra029966* | GGATGTTACTGAACTAGCCG | TAGTTGAGTACACCAGCAGA |
| *Bra024204* | GATCCGTCCGTCTACCCTTG | CCCTCCTGGCCGGTAAAATA |
| *Bra012640* | GGCATGGTTCTTGTTGTCTCC | TCAGCAAACCGTCTTTCCTTCTA |
| *Bra000575* | AACGGGTCTCCAGGTCAAAC | GAAAGCTTCGAGCTCCTCCA |
| *Bra004743* | AGGTCTCCTCCCTTCCAATCA | TGATCTGGTCGGTGTCGATG |
| *Bra018831* | AGAGAGTTTCACAGAAGGGA | AAAATCAGAATCCGGTCGTC |
| *Bra040182* | GGAGGAAAGATGGGTTGCTGA | AGCCCGGACAGTGACATAAG |
| *Bra022904* | CACATCCCGACCAGCTATCC | CCGGGTTTGATAGGACCGAG |

**Supplementary Table S2** Sequencing data and comparison statistics of each library.

| Sample | Raw reads | Clean reads | Mapped reads | Unique mapped reads |
| --- | --- | --- | --- | --- |
| S1 | 54125218 | 52178672 | 46442755 (89.01%) | 45253057 (86.73%) |
| S2 | 54650960 | 53446790 | 47363455 (88.62%) | 46267582 (86.57%) |
| S3 | 58405446 | 57173878 | 50627900 (88.55%) | 49523709 (86.62%) |
| B1 | 54307592 | 52879552 | 46647934 (88.22%) | 45488092 (86.02%) |
| B2 | 49747848 | 48488080 | 42692224 (88.05%) | 41538120 (85.67%) |
| B3 | 61677872 | 60491106 | 53241893 (88.02%) | 51887560 (85.78%) |
| L1 | 50121098 | 48655762 | 43670202 (89.75%) | 42083742 (86.49%) |
| L2 | 44695888 | 43543662 | 38980000 (89.52%) | 37644105 (86.45%) |
| L3 | 53080028 | 51060816 | 46199884 (90.48%) | 44398661 (86.95%) |

Raw reads, the number of reads in the original data; Clean reads, the number of high-quality reads filtered from the original data; Mapped reads, the number of reads that can be compared to the reference genome; Unique mapped reads, the fraction of mapped reads after duplicate reads are removed; S, stalk. B, flower bud. L, leaf. 1, 2, and 3 represent three biological replicates.
